# Potential Strategies for Kidney Regeneration With Stem Cells: An Overview

**DOI:** 10.3389/fcell.2022.892356

**Published:** 2022-05-02

**Authors:** Kenji Tsuji, Shinji Kitamura, Jun Wada

**Affiliations:** Department of Nephrology, Rheumatology, Endocrinology and Metabolism, Okayama University Graduate School of Medicine, Dentistry and Pharmaceutical Sciences, Okayama, Japan

**Keywords:** kidney regeneration, stem cell, *de novo* kidney, cell therapy, CKD

## Abstract

Kidney diseases are a major health problem worldwide. Despite advances in drug therapies, they are only capable of slowing the progression of kidney diseases. Accordingly, potential kidney regeneration strategies with stem cells have begun to be explored. There are two different directions for regenerative strategies, *de novo* whole kidney fabrication with stem cells, and stem cell therapy. De novo whole kidney strategies include: 1) decellularized scaffold technology, 2) 3D bioprinting based on engineering technology, 3) kidney organoid fabrication, 4) blastocyst complementation with chimeric technology, and 5) the organogenic niche method. Meanwhile, stem cell therapy strategies include 1) injection of stem cells, including mesenchymal stem cells, nephron progenitor cells, adult kidney stem cells and multi-lineage differentiating stress enduring cells, and 2) injection of protective factors secreted from these stem cells, including growth factors, chemokines, and extracellular vesicles containing microRNAs, mRNAs and proteins. Over the past few decades, there have been remarkable step-by-step developments in these strategies. Here, we review the current advances in the potential strategies for kidney regeneration using stem cells, along with their challenges for possible clinical use in the future.

## Introduction

The incidence of chronic kidney disease (CKD) patients continues to rise worldwide, and CKD has become an increasingly significant health burden. In end-stage kidney disease (ESKD), renal replacement therapy, including hemodialysis, peritoneal dialysis, and renal transplantation, is required. In 2010, 2.61 million people received dialysis or kidney transplantation, and it is estimated that the number will double by 2030 (Liyanage et al., 2015). Only a small number of people are able to receive a kidney transplant because of the small number of donors and the need to take immunosuppressive drugs after transplantation. Meanwhile, dialysis treatment does not completely replace the kidney’s role, leading to lower QOL and poor prognosis due to complications such as cardiovascular diseases. Despite recent technological developments, including wearable artificial kidneys (Gura et al., 2016) and bioartificial renal tubule assist devices (RADs) (MacKay et al., 1998) (Humes et al., 1999), it is still difficult to compensate for whole kidney function. Thus, the establishment of renal regeneration therapy is important, and strategies using stem cells might be one of the potential options to achieve this.

**FIGURE 1:**
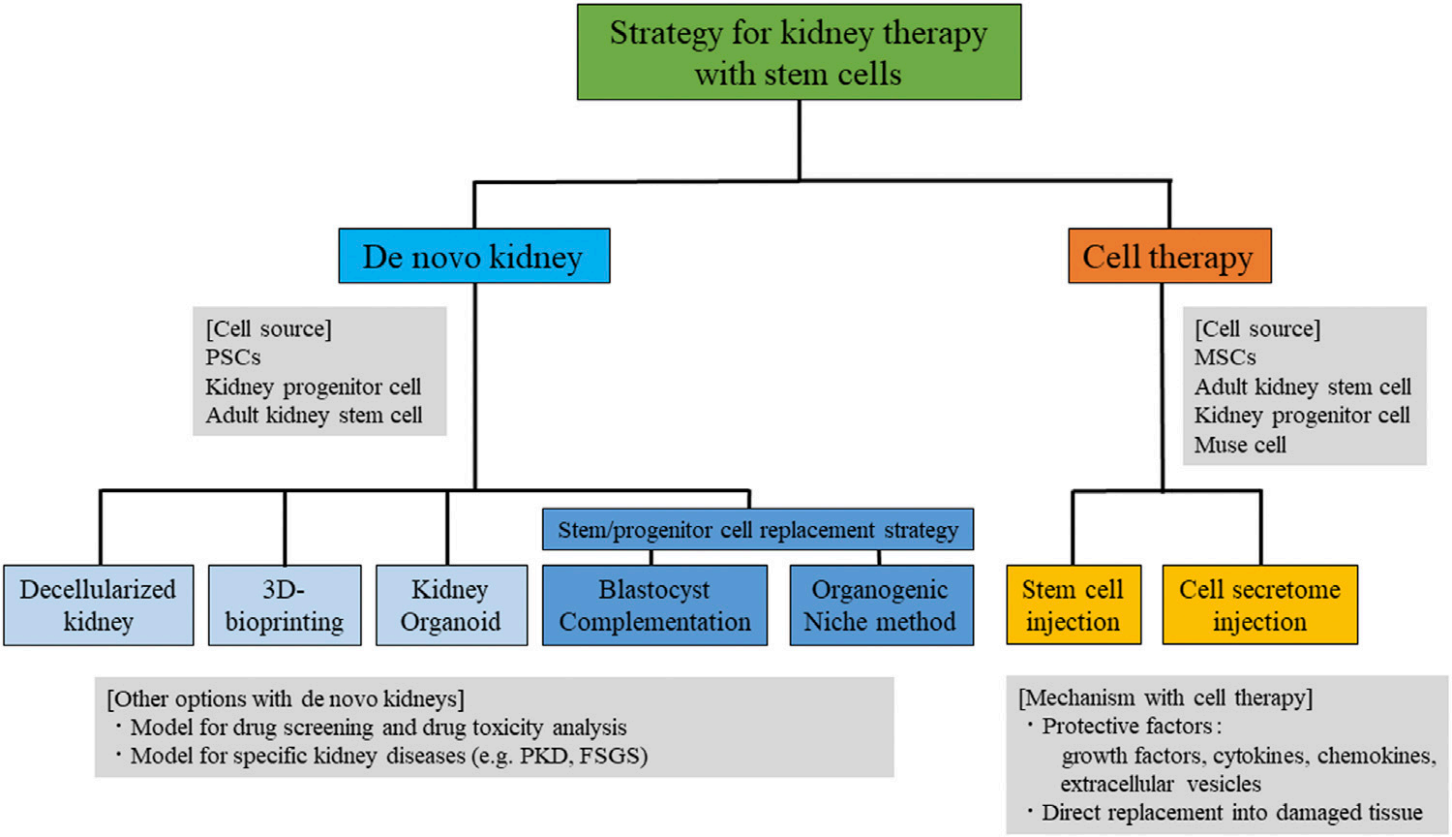
Summary of the strategies for kidney therapy with stem cells. PSCs, pluripotent stem cells; MSCs, mesenchymal stem cells; Muse, multi-lineage differentiating stress -enduring; PKD, polycystic kidney disease; FSGS, focal segmental glomerulosclerosis.

There are two directions for kidney regenerative strategies using stem cells: “rebuild” and “repair”. The “rebuild” strategy includes *de novo* kidney fabrication with stem cells and replacement by transplantation, while the “repair” strategy includes the induction of a native kidney repair system (Figure 1). There are several strategies for the fabrication of *de novo* kidneys, including 1) decellularized scaffold technology, 2) 3D bioprinting based on engineering technology, 3) kidney organoid fabrication, 4) blastocyst complementation technology, and 5) the organogenic niche method. Induction of a native kidney repair system includes, 1) injection of stem cells, such as mesenchymal stem cells (MSCs), nephron progenitor cells (NPCs), adult kidney stem cells, and multi-lineage differentiating stress enduring (Muse) cells, and 2) injection of protective factors secreted from stem cells. There have been remarkable step-by-step developments in both directions over the past few decades. Here, we summarize the current potential strategies for kidney regeneration using stem cells, as well as their remaining challenges for clinical use in patients with kidney diseases.

## De Novo Kidney Fabrication

### Decellularized Kidneys

The decellularization strategy is one of the options for *de novo* kidney fabrication. This method uses detergent, such as sodium dodecyl sulfate (SDS) and Triton X-100, to remove cells throughout the organs, and specific cells, such as stem cells, are then injected to differentiate into mature cells and engraft in the decellularized scaffold with the extracellular matrix (ECM). This concept was first applied in hearts (Ott et al., 2008) and then in lungs (Ott et al., 2010), livers (Mazza et al., 2015), and kidneys (Ross et al., 2009). For kidneys, mouse embryonic stem (ES) cells were injected into rat decellularized kidneys where differentiation into ES cell-derived blood vessels, glomeruli, and renal tubules was observed (Ross et al., 2009). Another group also applied this concept, wherein mouse ES cells were seeded on rat decellularized kidneys from the renal artery, showing uniform distribution of seeded cells in the vascular system and glomerular capillaries without signs of apoptosis (Bonandrini et al., 2014). Urine production from the recellularized kidneys was also investigated by injecting human umbilical vein endothelial cells (HUVECs) from renal arteries into decellularized rat kidneys and rat neonatal kidney cells from the ureter (Song et al., 2013). Another group also assessed urine production from recellularized kidneys with mouse ES cells, where repopulation occurred in the glomerular, vascular, and tubular structures, as well as urine production after implantation *in vivo* (Guan et al., 2015). The function of the decellularized scaffold ECM was evaluated in several studies. The expression pattern of ECM, such as of collagen type I and IV, laminin, heparan sulfate proteoglycan, and fibronectin, was investigated by using detergents such as 1% SDS and 1% Triton X-100 (Nakayama et al., 2010). Analysis of morphometry and resilience of ECM native vasculature with resin casting using electronic microscopy and pulse-wave measurements revealed that ECM preserved the microvascular morphology, morphometry, physiological function and growth factors, including VEGF and its receptor (Peloso et al., 2015). In another study, human induced pluripotent stem (iPS) cell-derived endothelial cells were injected through the renal artery and vein into decellularized kidneys, where continuous distributed cells along the vessel walls and fenestrae in the glomerular endothelium, but not in the vascular capillaries, were observed, suggesting site-specific endothelial cell specialization (Ciampi et al., 2019). More effective methods have also been explored. For example, use of pre-coated endothelial cells with subsequent implantation of human renal cells helped the vascularization (Huling et al., 2019). Despite the strong advantage of the availability of original native scaffolds, an important challenge is the difficulty of recellularization, because of its complexity.

### 3D-Bioprinting

The application of 3D bioprinting is another method of fabrication of *de novo* kidneys. Bioprinting is a technique involving the precise deposition of bioink (generally hydrogel containing cells) or biomaterial ink (without cells) layer-by-layer to construct complex tissues or organs (Fransen et al., 2021). There are several reports on the construction of tissues, such as ears (Mussi et al., 2019), but *de novo* whole kidney fabrication has yet to be reported. There are several challenges in the construction of whole kidneys, including the choice of cell types and biomaterials, and the complexity of the kidney structure, with its variety of cell types. Nevertheless, an important step has been reported, in which networks of vascularized constructs (Kolesky et al., 2016) (Kolesky et al., 2014), as well as proximal epithelial cells (Homan et al., 2016), were produced with 3D bioprinting. The same group further developed a dual channel kidney model with endothelium and epithelium channels (Lin et al., 2019). They applied a mixture of gelatin and fibrin as the ECM materials, and post-seeded the cells after the bioprinting. Another group reported a similar *in vitro* model of proximal tubules, without the need for post-seeding using bioink, where renal proximal tubular cells (PTECs) and HUVECs were included, respectively, in the decellularized ECM as natural-based materials (Singh et al., 2020). Although the product is far from a whole kidney fabrication, future development of the 3D printing system may allow multiple bioprinting with stem cells, which would allow the construction of more complex structures with several materials and/or cell types (McKee and Wingert, 2016). In addition, the product might be useful for *in vitro* drug nephrotoxicity testing and disease modeling. 3D bioprinting technology was also used in the kidney organoid model for high throughput drug nephrotoxicity analysis (Lawlor et al., 2021), where iPS cell-derived organoids were produced by the 3D bioprinting method, with higher reproducibility and in a shorter timeframe.

### Organoids

Creating kidney organs from pluripotent stem cells (PSCs) using self-organization technology is a major challenge. In general, self-tissue induction from PSCs should be based on knowledge of kidney development. Nephrons consist of three types of progenitor cells: NPCs, ureteric buds (UBs), and stromal progenitor cells. NPCs form tubular epithelial cells and glomeruli via mesenchymal-epithelial transition. UBs differentiate into collecting ducts through multiple branching. Stroma progenitor cells differentiate into interstitial cells. Several groups have reported the induction of NPCs via differentiation from PSCs. Taguchi et al. established NPCs by using a combination of multiple growth factors in multiple steps from iPS cells (Taguchi et al., 2014). Furthermore, by culturing NPCs with the spinal cord of a mouse embryo, they succeeded in creating kidney structures containing 3D glomeruli and tubules. Other groups have also established a protocol for constructing 3D nephrons, including glomeruli and tubules, using PSC-derived NPCs (Mae et al., 2013; Freedman et al., 2015; Morizane et al., 2015; Takasato et al., 2015). Among these, Takasato et al. reported the creation of renal structures from elements containing NPCs, UBs, and stromal progenitor cells, using a single protocol with iPS cells, although there was a defect in the normal UB branching structures (Takasato et al., 2015). Single-cell RNA sequencing analysis of kidney organoids generated by the Takasato protocol found no definitive collecting duct populations (Wu et al., 2018). Considering the distinct origin of nephrons and collecting ducts reported by Taguchi et al. (Taguchi et al., 2014), it is unlikely that the collecting ducts were co-induced by Takasato protocol. For the generation of the UB lineage, Taguchi et al. created UBs from ES cells and iPS cells, and cultured UBs with NPCs to create highly accurate kidney structures (Taguchi and Nishinakamura, 2017). Furthermore, by transplanting organoids into mice, the constructed organoids connected the vascular network of the host. Despite these step-by-step developments, several challenges remain. First, the organoid size currently being reported is too small to produce enough urine to maintain homeostasis in the body. Second, there is a need for trafficking with large blood vessels. Several groups have reported the construction of a vascular system by transplanting kidney organoids under the capsule of the kidney, but all of them were quite simple. Nevertheless, a recent study of kidney organoid creation with flow on millifluidic chips showed the expansion of vascular networks, as well as maturation of podocytes and tubular compartments, with higher levels of cell polarity and adult gene expression (Homan et al., 2019), indicating the progress of the vascular system in the kidney organoid. Third, the establishment of stromal progenitor cells from PSCs is still required. Fourth, maturity of the organoid remains an important problem. Taguchi et al. reported that the produced kidney organoids were comparable with E15.5 kidneys in a 7-days culture (Taguchi and Nishinakamura, 2017). In addition to fabrication by PSCs, another report exists of kidney organoids produced from adult kidney stem cells (Kitamura et al., 2015). By culturing clusters of adult kidney stem cells with multiple growth factors in Matrigel, the cell clusters differentiated into glomeruli, tubular epithelial cells, and collecting ducts, suggesting the presence of adult kidney stem cells with the capacity of differentiating into both NPC and UB genealogy. Although the final goal of using kidney organoids may be implantation as a functional kidney replacement, there are several options for their use, including drug screening, drug toxicity evaluation, and establishment of specific renal disease models for therapeutic drug screening. Indeed, the establishment of genetic model of polycystic kidney disease (PKD) using human PSC-derived kidney organoids was reported, where researchers showed cyst growth with several activations, such as cyclic AMP (Cruz et al., 2017). Tanigawa et al. established organoids created from NPHS1 missense mutations, where the podocyte foot process showed impaired slit diaphragm formation (Tanigawa et al., 2018). Furthermore, Gupta et al. identified the novel intrinsic repair mechanism using kidney organoids (Gupta et al., 2022). By using single-nuclear RNA sequencing analysis of kidney organoids after cisplatin exposure, they identified the transient up-regulation of Fanconi anemia complementation group D2 (*FANCD2*) and RAD51 recombinase (*RAD51*) during intrinsic repair, and the down-regulation of these genes in incomplete repair. The DNA ligase IV inhibitor SCR7 increased FANCD2-mediated repair and ameliorated injury in the organoids, suggesting a potential therapeutic pathway via FANCD2/RAD51. Collectively, organoid techniques may be useful for exploring specific disease and/or therapeutic mechanisms, as well as for therapeutic drug screening.

### Blastocyst Complementation

Blastocyst complementation is a method of implanting PSCs into undifferentiated germ cells of animals where a part of the tissue/organ is deficient, and deriving the deficient part from the implanted PSCs. By applying this strategy to a Pdx1 deficient pancreatogenesis-disabled rodent model, a PSC-derived pancreas was created (Kobayashi et al., 2010; Yamaguchi et al., 2017). The established pancreas was largely derived from the implanted PSCs. This strategy was also applied to the kidneys. By injecting ES cells into the blastocysts of mice lacking the Sall1 gene, which is essential for metanephric mesenchymal development, Usui et al. succeeded in PSC-derived kidney production; the glomerulus and tubular cells were derived from ES cells, while other collecting duct and vascular cells were derived from the host (Usui et al., 2012). Furthermore, interspecies generation was also reported. Goto et al. transplanted mouse-derived ES cells into Sall1-deficient rats in an interspecies experiment, and succeeded in mouse PSC-derived kidney production, where the glomerular and tubular epithelium were entirely composed of mouse PSC-derived cells with normal formation of the ureter-bladder junction (Goto et al., 2019), indicating the possibility of using this strategy in a xenogeneic host, for example, using human PSCs. Indeed, human PSCs were implanted into pig embryos by blastocyst complementation, but the chimerism was quite low, and was insufficient for organ development (Wu et al., 2017). The establishment of human iPS cells capable of forming enough chimeras, or combining the technique with an inhibition technique of apoptosis, might be required. Another problem of blastocyst complementation is the ethical concerns of chimerism, due to the possible presence of human-derived cells in neural or germline cells (Hermeren, 2015). Thus, there is a need for techniques that can differentiate PSCs only into specific organs by genetic manipulation. Since only the glomeruli and tubular cells were from implanted PSCs in the Sall1-deficient animals, another step will be required to achieve whole kidney replacement from an implanted PSCs origin.

### Organogenic Niche Method

The organ niche method uses the organ niche of later stage embryo kidneys by applying the concept of blastocyst complementation where organ progenitor cells are implanted at the site and timing of organ development and are cultured *ex vivo* in a growing fetal system to differentiate into organs. Yamanaka et al. applied this strategy in their experiments (Yamanaka et al., 2017). They implanted mouse NPCs into the nephrogenic zone of a Six2-cre inducible diphtheria toxin receptor (iDTR) mouse model, and diphtheria toxin (DT) treatment induced ablation of DTR-positive Six2 expressing host metanephric mesenchymal cells. The implanted NPCs replaced the host metanephroi and differentiated into glomerular and tubular cells, while the collecting duct and vascular cells were derived from the host mice. In addition, they succeeded in producing similar kidney structures using rat-derived NPCs in Six2-cre iDTR mice, achieving interspecies chimerism. Since the DT induces apoptosis in human cells, DT method may not be useful for human iPS cells. Therefore, they used the tamoxifen-inducible ablation model and succeeded in achieving urine production with the transplantation of NPCs in mouse embryos in uterine (Fujimoto et al., 2020). They found that human iPS-derived renal vesicles, which were connected to the host UBs, did not differentiate into mature nephrons like mouse or rat NPCs. Furthermore, they developed a new system, the stepwise peristaltic ureter (SWPU) system, for building a ureter system while solving several important problems in the construction of a urine excretion pathway and continuous growth of a newly generated kidney (Yokote et al., 2015). They transplanted the rodent metanephroi with bladders (cloacas) into host rodents, and then connected the host animal’s ureter to the cloaca’s bladder after the cloaca’s growth. This system avoided hydronephrosis and allowed the cloacas to develop in the host animals. They performed a similar experiment using pig kidneys, achieving 3 cm metanephroi growths and production of urine. With the combination of these strategies, it might be possible to develop a functional *de novo* kidney of adequate size and with a urine excretion system. However, there are still several problems to be solved, including chimeric problems similar to those of the blastocyst complementation strategy. Innovative breakthroughs in whole kidney replacement by implanted PSCs, without the distribution of neural or germline systems, would be required.

## Stem Cell Therapy

### Administration of Stem Cells

Stem cell administration is an option to induce regenerative mechanisms. There are two mechanisms for implanted stem cells: replacement of injured tissue through migration, differentiation, and engraftment to the damaged site, and paracrine effects from implanted stem cells. The advantage of stem cell injection is their homing effect (Liesveld et al., 2020); implanted stem cells migrate to the injured tissue and may contribute to local paracrine effects. There are plenty of published reports on the regenerative effects of MSC intravenous administration into rodent models of acute kidney injury (AKI), induced by drug or ischemia/reperfusion (I/R) injury and CKD models, such as 5/6 nephrectomy, diabetes nephropathy, and unilateral ureteral obstruction (Tsuji and Kitamura, 2015). While MSC-derived cell engraftment has been reported as a direct replacement mechanism (Broekema et al., 2005), the evidence suggests that the major trophic effect can be attributed to paracrine effects. MSCs can be obtained from several tissues, including bone marrow, adipose tissue, and the umbilical cord. Despite possible heterogeneities due to MSC origins, each of these MSCs have been reported to be reno-protective (Tsuji et al., 2018). Regarding kidney specific stem cells, Kitamura et al. established adult kidney stem cells (Kitamura et al., 2005), and the administration of the cells ameliorated renal function in an I/R-induced AKI model through the replacement of injured tissue. In addition, several reports have demonstrated PSC-derived NPC administration, where preserved renal function was mediated in drug or I/R-induced AKI and 5/6 nephrectomy-induced CKD (Harari-Steinberg et al., 2013; Imberti et al., 2015; Toyohara et al., 2015). Furthermore, administration of Muse cells, nontumorigenic endogenous pluripotent-like stem cells that can be collected from various organs, was reported to ameliorate an Adriamycin-induced focal segmental glomerulosclerosis model through homing to damaged glomeruli and differentiation into glomerular cells (Uchida et al., 2017). Collectively, stem cell administration may have therapeutic potential for both AKI and CKD via both direct and indirect mechanisms.

### Administration of Stem Cell Secretome

Since the paracrine effect of stem cell therapy may make the predominant contribution in stem cell therapy, the secretome of stem cells and regenerative mechanisms are of interest. The secretome of MSCs (Tsuji et al., 2018), as well as the regenerative mechanisms (Tsuji and Kitamura, 2015), have been well summarized. Previous reports revealed that paracrine mechanisms include cell proliferation, suppression of apoptosis, regulation of inflammation, angiogenesis, and dedifferentiation of tubular cells (Tsuji et al., 2020). It is believed that various factors work orchestrally to mediate these mechanisms. MSCs may secrete growth factors, chemokines and extracellular vesicles (EVs) containing microRNAs (miRNAs), mRNAs, and proteins (Tsuji et al., 2020). As growth factors, MSCs secrete hepatocyte growth factor (HGF), EGF-like growth factor (EGF), basic fibroblast growth factor (bFGF), and vascular endothelial growth factor (VEGF), all of which have been reported to be reno-protective (Tsuji and Kitamura, 2015). In addition, accumulating evidence has revealed that EV-containing miRNAs from MSCs are essential contributors in stem cell therapy. Trophic miRNAs from MSCs include miR-21, miR-199, the Let-7 family, and miR-30, which may silence their target genes through binding to the 3′-UTR. It has also been reported that these secretomes may be modified by several stimuli, including hypoxia, inflammatory stimuli, and modified culture conditions (Tsuji et al., 2018). Since EVs may work as a natural drug delivery system by transporting several factors into distant organs with high stability, the treatment of EVs from stem cells with the manipulation of specific miRNAs might be another promising option.

## Conclusion

Here, we summarize two directions “rebuild” and “repair”, for the use of stem cells. For both, there remains several challenges for clinical use. Regarding the “rebuild” methods, vascular invasion into *de novo* kidneys, adequate kidney size for urine production, induction of podocytes that do not exhibit urinary proteins, and kidney production via a detailed mechanism by which urine flows into the renal tubules and reabsorbs important substances are all required. Due to these complexities in both structure and function, the rebuilding of kidneys is difficult compared to other organs. Nevertheless, considering the rapid progression over the past few decades, “rebuild” techniques may be possible in the future. Regarding “repair”, we still need to elucidate the kidney regenerative mechanisms so that we may activate these mechanisms using stem cells or stem cell-mediated secretomes. Despite these challenges, regenerative treatment using stem cells is a promising strategy for reno-protection.
